# Exploring Variations in Etiology and Clinical Presentations of Kounis Syndrome Across Pediatric and Adult Populations: A Comprehensive Review

**DOI:** 10.7759/cureus.56249

**Published:** 2024-03-16

**Authors:** Houssam Eddine Youcefi, Alara Abu Saadeh, Gürkan Karaca, Ali Kimiaei, Seyedehtina Safaei, Adnan Kaya

**Affiliations:** 1 Cardiology, Bahçeşehir University, Istanbul, TUR

**Keywords:** coronary spasm, allergic angina, allergy and immunology, acute coronary syndrome, kounis syndrome

## Abstract

Kounis syndrome (KS), recognized as a rare yet significant form of acute coronary syndrome precipitated by allergy-mediated mechanisms, poses diagnostic challenges due to its varied clinical presentations and under-recognition. Despite its relevance across diverse populations, comprehensive insights into age-specific characteristics and management remain limited.

The analysis of 420 studies yielded a total of 466 case reports of Kounis syndrome, categorized into pediatric (n = 31) and adult (n = 435) populations. After rigorous screening, 330 adult and 20 pediatric case reports were included for further analysis. Triggering factors were identified, with drugs (other) being the most prevalent in both groups. The breakdown of triggering factors, such as drugs (antibiotics), bee/wasp stings, and contrast media, was elucidated. Variations in presenting symptoms, diagnostic investigations, and treatment modalities between pediatric and adult populations were observed. Notably, all pediatric cases were diagnosed with subtype I Kounis syndrome and demonstrated favorable outcomes without any reported fatalities, whereas adult cases exhibited a broader range of Kounis subtypes. Mortality was recorded solely in adult case reports, with no fatalities reported among pediatric cases. These findings underscore the importance of understanding the nuances in the clinical presentation and management of Kounis syndrome across different age groups.

## Introduction and background

Kounis syndrome (KS), also known as allergic angina, is a rare form of acute coronary syndrome triggered by allergy-mediated processes [1]. It was first described by Kounis and Zavras in 1991 as the concomitance of acute coronary syndromes with conditions involving mast cell activation, encompassing a spectrum from coronary spasm to myocardial infarction [2]. Various conditions can induce KS, including drugs, environmental exposures, bee or wasp stings, foods, and coronary drug-eluting stents [3]. There are three main variants of KS, including allergic vasospastic angina, allergic myocardial infarction, and allergic stent thrombosis. 

While there is a need for large prospective trials to determine its prevalence and exact incidence, in the only prospective study conducted, including 138,911 emergency admissions, the incidence of Kounis syndrome was reported at 19.4 per 100,000 among all admissions and 3.4% among allergy patients [4]. Recently, KS gained clinical significance with observations across different races, age groups (from two to 90 years old), and geographical locations [3]. Although initially considered rare, its relative infrequency in the literature and clinical practice is attributed to missed, unrecognized, and/or undiagnosed cases [3], due to even more complexity in diagnosing pediatric patients, emphasizing the importance of increased awareness to prevent oversight of these patients. 

The limited literature addressing the specific characteristics of KS in both pediatric and adult populations highlights the need for a comparative review. By examining how this syndrome manifests and progresses in diverse age groups, we could provide valuable insights into potential age-related differences in presentation, triggers, and outcomes, paving the way for age-specific diagnostic and therapeutic strategies.

In this review article, we aimed to fill crucial gaps in our understanding of Kounis syndrome through three key objectives. Firstly, we investigated age-specific etiological factors contributing to KS. Secondly, we compared clinical presentations and manifestations of KS in pediatric and adult patients. Lastly, we examined variations in management approaches and outcomes based on age, with the goal of informing more precise and effective care strategies for KS across diverse age groups.

## Review

Materials and methods

Eligibility Criteria, Study Selection, and Screening Process

The eligibility criteria for inclusion were defined to ensure the selection of pertinent literature. Articles had to encompass case reports or case series written in English detailing the clinical presentation of patients diagnosed with Kounis syndrome in either pediatric or adult populations. Crucial information, such as patient age, triggering factors, presenting symptoms, diagnostic investigations (including electrocardiography (ECG), echocardiography, cardiac enzyme levels, and coronary angiography), treatment administered, and outcomes, needed to be explicitly available within the article. Articles were excluded from the analysis under the following criteria: if the language was other than English, if the article was unable to be retrieved, if there was incomplete information regarding patient status, or if they were review articles, meta-analyses, or systematic reviews. The scope was narrowed to articles containing comprehensive data aligned with the research objectives, excluding those lacking essential details or not meeting the specified criteria. This rigorous selection process aimed to gather high-quality and relevant evidence for the review while maintaining a focus on the clinical characteristics, risk factors, management strategies, and outcomes of Kounis syndrome across different age groups.

**Figure 1:**
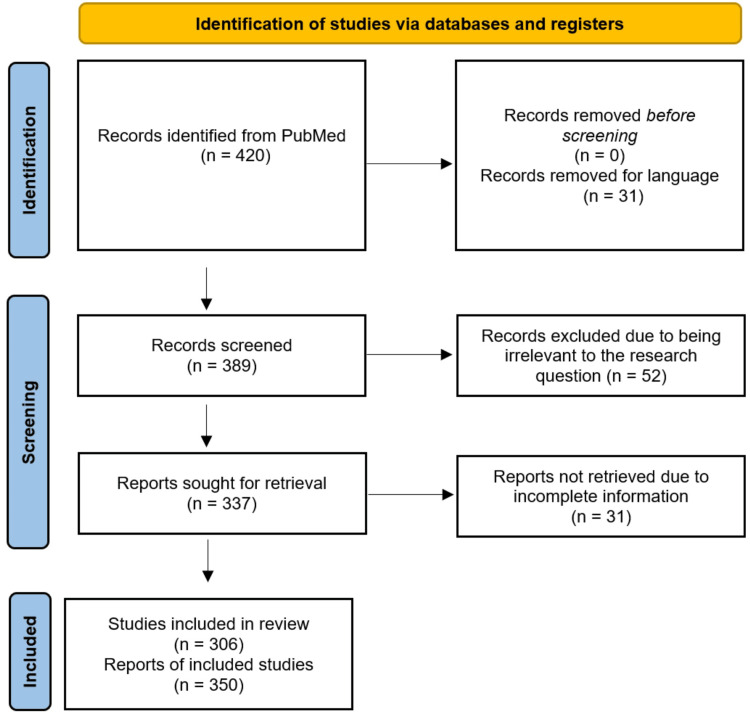
A flow chart illustrating the procedure and findings of the literature search, in accordance with the Preferred Reporting Items for Systematic Reviews and Meta-analyses (PRISMA) guidelines.

The study selection and screening process followed a meticulous two-stage approach to ensure the review included the most pertinent and appropriate articles. Initially, a comprehensive search strategy was executed across PubMed, covering literature from inception to September 2023, utilizing specific Medical Subject Headings (MeSH) terms such as "Kounis Syndrome," "Adult," "Pediatrics," "Case Reports," and "Case Series." Boolean operators (e.g., AND, OR, NOT) were also utilized to refine the search strategy and ensure comprehensive coverage. This initial stage involved screening the titles and abstracts of the identified articles to assess their relevance to the research question and eligibility criteria. Following this, a rigorous full-text assessment of selected articles was conducted, focusing on the inclusion criteria and extracting detailed information. Any discrepancies or disagreements between reviewers during these stages were resolved through discussion to maintain consistency and rigor in the selection process. The literature search process and outcomes, following Preferred Reporting Items for Systematic Reviews and Meta-analyses (PRISMA) guidelines, are depicted in Figure 1.

Data Extraction

During the data extraction phase, a meticulous approach was adopted to retrieve essential variables from each selected article that contributed to a comprehensive understanding of Kounis syndrome across different populations. These variables encompassed detailed information such as the age group and specific age of patients, including any gender-related data mentioned in the articles. Additionally, data were extracted regarding the triggering factors that induced Kounis syndrome, the presence of specific symptoms like dyspnea, angina, or rash/urticaria, as well as findings from diagnostic investigations including electrocardiography (ECG), echocardiography, cardiac enzyme levels, eosinophilia, coronary angiography, and echocardiographic findings at discharge.

Moreover, the extraction process focused on identifying the Kounis syndrome subtype (types I, II, or III) presented in each case, the treatment modalities administered, and whether there was a reported mortality outcome (yes or no). Each of these data points was collected and organized from the selected articles, ensuring a comprehensive and detailed extraction of relevant information pertaining to clinical characteristics, diagnostic findings, management strategies, and patient outcomes associated with Kounis syndrome across pediatric and adult populations.

Results

Literature Search

From the database search, we obtained 420 studies. These studies were reviewed for potential relevance. Case reports and case series study designs were both included in the analysis; hence, the total number of case reports obtained was 466. We further divided the available studies into two groups: pediatric (n = 31) and adult (n = 435) patients. Through abstract and full-text screening, 116 case reports were excluded in accordance with the exclusion criteria (articles in languages other than English, articles with incomplete information, and systematic reviews). The final number of adult and pediatric case reports included in the analysis was 330 and 20, respectively.

Comparison of Triggering Factors in Both Groups

The identified triggering factors were categorized into distinct groups, namely drugs (antibiotic), drugs (other), food, vaccine, bee/wasp sting, other animal/insect bite, contrast media, unknown, and others (Table 1). Notably, the most prevalent triggering factor in both adults and pediatric patients was drugs (other), which included a variety of drugs, not including antibiotics, with a rate of 29.1% and 25.0%, respectively. The detailed breakdown of drugs (other) is available in Table 2.

**Table 1:** The frequency of trigger factors of Kounis syndrome in both populations.

Triggering factor	Pediatric case reports	Adult case reports
Drugs (antibiotics)		
Amoxicillin	3	29
Cephalosporin	0	23
Clindamycin	1	1
Metronidazole	1	0
Drugs (other)	5	97
Wasp and bee stings		
Bee	3	29
Wasp	2	18
Contrast media	0	23
Vaccine	0	8
Food	0	29

**Table 2:** List of drugs that were the culprits in Kounis syndrome. NSAIDs: non-steroidal anti-inflammatory drugs.

Adults (n = 98)	Pediatric (n = 5)
Anesthetics (Bupivacaine, Cisatracurium, and Lidocaine)	Anti-cholinergic (atropine)
Anti-arrhythmic (Amiodarone)	Non-depolarizing neuromuscular blockers (vecuronium)
Anti-cholinergic (Anisodamine)	Opioids (tramadol)
Anti-emetic (Chlorphenamine, Domperidone, and Ondansetron)	Sedative (midazolam)
Anti-fungal (Fluconazole)	Other (epinephrine)
Anti-psychotics (Ziprasidone)	-
Chemotherapeutics (Capecitabine, Carboplatin, Cisplatin, Epirubicin, Oxaliplatin, Paclitaxel)	-
Monoclonal antibodies (Daratumumab, Rituximab)	-
NSAIDs (Aspirin, Celecoxib, Diclofenac, Ibuprofen, Ketorolac, Loxoprofen, Naproxen sodium, and Nimesulide)	-
Non-depolarizing neuromuscular blockers (Rocuronium, Vecuronium)	-
Opioids (Fentanyl, Morphine, and Tramadol)	-
Proton pump inhibitors (PPIs) (Lansoprazole, Omeprazole, and Pantoprazole)	-
Sedative (Midazolam)	-
Spasmolytic (Phloroglucinol, Urapidil)	-
Other (Allopurinol, angiotensin-converting enzyme inhibitor, Epinephrine, Isotretinoin, Methimazole, Paracetamol, Progesterone, Protamine sulfate, Succinylated gelatin, Sugammadex, Bivalirudin, Clopidogrel, Hyoscine butylbromide, Low-molecular-weight heparin)	-

In the adult group, the other triggering factors were, from most prevalent to least prevalent in reference to the studies that were included in this study, drugs: antibiotics (21.1%), bee/wasp stings (14.2%), food (8.8%), other (7.9%), contrast media (6.7%), insect bites (6.1%), unknowns (3.6%), and vaccines (2.4%). The consequent triggering factors in the pediatric population were as follows, from most prevalent to least prevalent: bee/wasp stings (25%), drugs (antibiotics) (25%), insect bites (15%), and others (10%).

Category 1: Drugs Excluding Antibiotics

The detailed analysis of pediatric case reports that belonged to the drugs (other) group was as follows: atropine (n = 1), epinephrine (n = 1), midazolam (n = 1), rocuronium (n = 1), and tramadol (n = 1). The most frequent cause of Kounis syndrome in the adult population was also presented in the drug (other) category, which included various drugs such as NSAIDs, immunosuppressants, PPI, lidocaine, and midazolam (Table 2).

Category 2: Drugs Including Antibiotics

When considering antibiotics, it was the triggering factor in 71/330 of adult case reports and 5/20 of pediatric studies that were included in this study. The most reported antibiotic affecting adults was amoxicillin in 29/71 of studies reporting antibiotic-induced Kounis syndrome in the adult population. Likewise, in the pediatric population, 3/5 of the studies reporting antibiotic-induced Kounis syndrome were due to amoxicillin with clavulanic acid. Other studies of pediatric patients also reported cases with clindamycin and metronidazole.

Category 3: Wasp/Bee Stings

Bee and wasp stings were placed in a separate category from other animal and insect stings due to their increased frequency in the etiology of Kounis syndrome in the literature. Wasp sting or bite was the causative factor in 47 of the adult group studies and in five of the pediatric groups. Other insect bite cases were also found and included snakes, spiders, Hymenoptera, ant bites, and caterpillars.

Category 4: Contrast Media

No cases of contrast media-induced Kounis syndrome were reported in the pediatric population; however, 23/330 of the studies we examined in the adult population were due to contrast media. Contrast agents included gadolinium, iohexol, loperamide, and ioversal.

Category 5: Vaccine

No cases of vaccine-induced Kounis syndrome were reported in pediatric patients; however, 8/330 of the case reports we examined in the adult population were due to vaccines. The only vaccine reported to cause Kounis syndrome in the literature that we examined was the COVID-19 vaccine.

Category 6: Food

No cases of food-induced Kounis syndrome were reported in the pediatric population; however, 29/330 of the case reports we examined in the adult population were due to food substances. This included fruits such as kiwi, pineapple, and banana; fish-related foods were also a recurrent culprit of Kounis syndrome, such as tuna, shellfish, and sushi.

Category 7: Others

The ‘others’ section included a variety of triggering factors that did not fit in any other category. It comprised 7.9% of the adult population case reports and 10% of the pediatric case reports. This included branched-chain amino acids, nickel, drug-eluting stents, latex, marijuana, nicotine patches, anti-snake venom, platelet transfusions, and postpartum.

Comparison of Presenting Symptoms and Subtypes

The comparison of diagnostic investigations is summarized in Table 3.

**Table 3:** Break down of diagnostic investigations obtained from case reports.

Diagnostic investigations	Pediatric case reports (n = 20)	Adult case reports (n = 330)
Electrocardiography ischemic changes	19	295
Elevated cardiac enzymes	17	218
Pathologic findings on coronary angiography	1	160
Reduced ejection fraction on echocardiography	5	55
Eosinophilia	7	54

Shortness of Breath, Angina and Urticaria 

When investigating the presenting symptoms between pediatric and adult patients, we found that 7/20 and 151/330 presented with shortness of breath. Two hundred and fifty-one adults presented with angina, as well as 14 pediatric case reports. 9/20 of pediatric patients also presented with a rash. 

ECG 

ECG changes were noticed in 19/20 of patients in the pediatric population and in 295/330 of the adult population.

Cardiac Enzymes 

An elevation in cardiac enzymes was seen in 17/20 of pediatric case reports. In adult patients, an increase in cardiac enzymes also showed a predominance (218/330).

Angiography 

In the pediatric population, 11/20 of the case reports reported normal arteries on angiography, 8/20 did not have an angiography, and only one case reported stenosis in the coronary arteries. In the adult case reports, pathologic coronary angiography was found in 160/330 cases.

Echocardiography 

About 5/20 of patients were reported to have an EF<50% in the pediatric population; however, 19/20 had an EF>50% at the time of discharge. 55/330 of the adult case reports also presented with EF<50%.

Kounis Subtype 

About 100% of the pediatric population was reported to be diagnosed with subtype I of Kounis syndrome. While the most common subtype of Kounis syndrome in adults was type I, there were also cases reported of type II (67/330) and type III (28/330).

Treatment Modalities 

All pediatric patient case reports were treated with medical management. This included, but was not limited to, antihistamines, corticosteroids, aspirin, Ca channel blockers, and beta blockers.

Mortality Rate

Pediatric case reports of Kounis syndrome documented no fatalities. Nonetheless, instances of mortality (16/330) were recorded in case reports of Kounis syndrome in adults.

Discussion

Kounis syndrome is defined as the concurrent occurrence of acute coronary syndrome and an allergic hypersensitivity reaction associated with the release of inflammatory cells such as mast cells, platelets, and eosinophils [3]. It can be divided into three subtypes: type I (myocardial infarction with non-obstructive coronary arteries), type II (allergic myocardial infarction), and type III (stent thrombosis) (see Table 4) [3,5]. As we have reported in our investigation of the literature, there is no set data on what can and can’t cause Kounis syndrome. The list is long and varied, ranging from antibiotics, analgesics, immunosuppressant drugs, foods such as fruit and fish, allergic reactions to bee and wasp stings, contrast agents, and drug-eluting stents.

**Table 4:** Subtypes of Kounis syndrome.

Type	Description
Type I: coronary spasm	Endothelial cell dysfunction
	Normal arteries on angiography
	No history of coronary artery disease
	± elevated cardiac enzymes
Type II: myocardial infarction	Pre-existing atheromatous disease
	Plaque erosion or rupture on coronary angiogram leading to occlusion of coronary arteries
Type III: stent thrombosis	History of previous stent implantation
	Thrombosis composed of eosinophils and mast cells forms over the stent and occludes the coronary arteries

The most common antibiotics that were noted to cause Kounis syndrome in pediatric case reports were amoxicillin and clavulanic acid [6,7]. Clindamycin and metronidazole were also reported to cause Kounis syndrome in pediatric patients [8,9]. In the adult case reports, antibiotics such as vancomycin, cephalosporins, amoxicillin and clavulanic acid, clarithromycin, sulbactam-ampicillin, gemifloxacin, and penicillin were among the common antibiotics that lead to Kounis syndrome [7]. Atropine, epinephrine, midazolam, rocuronium, and tramadol were also reported as other drugs that led to Kounis syndrome in the pediatric case reports [10-14]. Across the 330 case reports that we finalized on Kounis syndrome in the adult population, a variety of drugs were the causative agents of Kounis syndrome, including NSAIDs, immunosuppressants, neuromuscular blocking agents, antipsychotics, and inhaled anesthetics (see Table 2). Bee and wasp stings were also common triggering factors across the literature in both pediatric and adult patients. Other triggering factors, such as spiders and scorpion bites, were also reported in the pediatric population and adult population [15-17]. One case also reported a 17-year-old male who suffered from Kounis syndrome due to taking branched-chain amino acid supplements [16]. We also noted that trigger factors such as contrast agents and food (fish-related products and fruits especially) were present in adult case reports but not in pediatric cases [18-66].

Kounis syndrome symptoms can resemble an acute coronary syndrome presentation with angina pectoris, dyspnea, stridor, drowsiness, syncope, and acute pulmonary edema [5]. In addition to the common symptoms of acute coronary syndrome, some patients also present with urticaria. While reviewing the literature, we noted that most adult case reports reported urticaria; likewise, in pediatric case reports, 45.0% presented with rash or urticaria [10,14,16,19,67,68]. Typical angina was the most common type of presenting angina for Kounis syndrome in both adult and pediatric case reports. Dyspnea was also a common presenting symptom in both age groups; 65% of pediatric case reports presented to the emergency department with dyspnea. Loss of consciousness was present in one pediatric patient in the literature [11]. Additionally, three out of the 20 case reports in the pediatric age group developed pulmonary edema at the time of presentation [13,14,69,70]. Other presentations in the adult case reports include seizures, periorbital edema, kidney failure, cardiogenic shock, ventricular fibrillation, and cardiac arrest [71-82]. Whether these presentations were directly related to Kounis syndrome or the specific triggering factor is not clear.

Diagnosing Kounis syndrome can often be a challenging task, especially when it comes to pediatric patients, as myocardial infarction is not commonly considered a potential cause for typical angina symptoms. It is of utmost importance to diagnose Kounis syndrome accurately and promptly to achieve a more favorable outcome. The diagnosis is primarily based on the clinical signs as well as the findings from electrocardiography (ECG), echocardiography, and angiography. Furthermore, elevated levels of cardiac enzymes, tryptase, and eosinophilia can also serve as indications of Kounis syndrome. In all reported cases involving pediatric patients, ischemic changes (ST elevation and ST depression) were observed on the ECG, except for one case where a 15-year-old patient with Kounis syndrome, due to a wasp sting, displayed normal findings on the ECG [34,40,64-66,72,83-90]. Similarly, in the case of the adult case reports, it was also observed that ischemic changes were noted on the electrocardiogram (ECG). Nevertheless, some case reports also documented normal findings on the ECG. 

Echocardiography is useful in detecting wall motion abnormalities and ejection fractions both at presentation and before discharge. 50% of patients in the pediatric case reports related to Kounis syndrome presented with EF>50% [6-8,10,13,67-69,83,91]. Despite some pediatric patients presenting with EF<50%, most patients had EF>50% at discharge [11-12,14,16-17]. Hypokinesia was also a common finding on echocardiography in the pediatric population [6,9,19,68]. Cardiac enzymes were elevated in 17 out of the 20 pediatric case reports that we investigated. Coronary angiography is another crucial tool for the diagnosis of Kounis syndrome. In the 20 pediatric case reports, 11 patients presented with normal coronary arteries, eight patients had no report of angiography, and one patient, a two-year-old male, was found to have stenotic coronary arteries on angiogram [13]. Hence, type I is the most common Kounis syndrome variant reported in the pediatric case reports of Kounis syndrome available currently in the literature. Type I was also the most frequently encountered variant among adult case reports in the literature. Type II and type III were reported in multiple adult case reports; however, no case reports of type II or III in the pediatric population were reported to our knowledge.

Medical management of Kounis syndrome includes aspirin, nitroglycerin, heparin, beta blockers, calcium channel blockers, antihistamines, corticosteroids, and epinephrine [92]. Placement of drug-eluting stents is also used in the management of type II and type III variants of Kounis syndrome. Calcium channel blockers, antihistamines, and prednisone were the most commonly used medical management strategies in pediatric case reports presented in the literature. Three out of 20 case reports of pediatric patients resolved spontaneously. Medical management in both age groups yielded similar results; however, due to the occurrence of types II and III, adult Kounis syndrome was also managed with drug-eluting stents for type II and thrombus aspiration with new stent placement in type III.

There are no reported deaths in the literature due to Kounis syndrome in the pediatric population. Sixteen out of the 330 case reports that we investigated of adult patients reported death due to Kounis syndrome or its complications [27,93-102].

## Conclusions

In the intricate realm of Kounis syndrome, where acute coronary events intertwine with allergic reactions, this review unveils a complex tapestry of triggers, presentations, and management strategies across pediatric and adult populations. The diversity of triggers, from medications to insect stings, demands heightened vigilance for accurate diagnosis, especially in pediatric cases where myocardial infarction might not immediately come to mind. Diagnostic challenges persist, emphasizing the need to integrate clinical signs with various imaging and laboratory findings. While therapeutic approaches converge on medications like antihistamines and corticosteroids, nuances arise in interventions, with adults often requiring invasive procedures like drug-eluting stents. Notably, pediatric cases show a lack of reported fatalities, hinting at potentially better prognoses in this age group compared to adults, where complications have led to fatal outcomes. This comprehensive analysis underscores the call for heightened awareness, tailored diagnostic pathways, and refined therapeutic interventions specific to distinct age cohorts, aiming to enhance care and outcomes for those affected by Kounis syndrome.
